# Ensemble heterogeneity mimics ageing for endosomal dynamics within eukaryotic cells

**DOI:** 10.1038/s41598-023-35903-0

**Published:** 2023-05-31

**Authors:** Nickolay Korabel, Alessandro Taloni, Gianni Pagnini, Viki Allan, Sergei Fedotov, Thomas Andrew Waigh

**Affiliations:** 1 Department of Mathematics, The University of Manchester, Manchester, M13 9PL UK; 2 CNR-Consiglio Nazionale delle Ricerche, Istituto dei Sistemi Complessi, via dei Taurini 19, 00185 Rome, Italy; 3 BCAM-Basque Center for Applied Mathematics, Mazarredo 14, 48009 Bilbao, Basque Country Spain; 4 Ikerbasque-Basque Foundation for Science, Plaza Euskadi 5, 48009 Bilbao, Basque Country Spain; 5 School of Biological Sciences, The University of Manchester, Manchester, M13 9PT UK; 6 Biological Physics, Department of Physics and Astronomy, The University of Manchester, Manchester, M13 9PL UK

**Keywords:** Biological physics, Biophysics

## Abstract

Transport processes of many structures inside living cells display anomalous diffusion, such as endosomes in eukaryotic cells. They are also heterogeneous in space and time. Large ensembles of single particle trajectories allow the heterogeneities to be quantified in detail and provide insights for mathematical modelling. The development of accurate mathematical models for heterogeneous dynamics has the potential to enable the design and optimization of various technological applications, for example, the design of effective drug delivery systems. Central questions in the analysis of anomalous dynamics are ergodicity and statistical ageing which allow for selecting the proper model for the description. It is believed that non-ergodicity and ageing occur concurrently. However, we found that the anomalous dynamics of endosomes is paradoxical since it is ergodic but shows ageing. We show that this behaviour is caused by ensemble heterogeneity that, in addition to space-time heterogeneity within a single trajectory, is an inherent property of endosomal motion. Our work introduces novel approaches for the analysis and modelling of heterogeneous dynamics.

## Introduction

In eukaryotic cells, endosomes form a highly dynamic and heterogeneous network which is comprised of various forms of early and late endosomes, each with distinct structures and functions^1^. The endosomal network plays a major role in sorting and transporting proteins and lipids that are taken in from the cell surface and need to be delivered to lysosomes for degradation. Early endosomes, formed by the budding of clathrin-coated vesicles, are characterized by their high levels of Rab5, a small GTPase protein. Late endosomes, on the other hand, are characterized by lower levels of Rab5 and higher levels of Rab7^2^. Rab5 early endosomes move towards the cell nucleus via dynein^3^, fuse with one another, increase in size and change their membrane composition. The open question remains: how does the heterogeneity of the endosomal network influence endosomal dynamics? Here we address this question.

It is known for a long time that endosomal movement is anomalous^4–9^. Anomalous diffusion is defined by the non-linear growth of the mean squared displacement (MSD), i.e., $\left\langle r^2(t) \right\rangle \sim t^{\alpha }$ ($\alpha \ne 1$)^10,11^. Such non-linear scaling is related to the breakdown of the central limit theorem. The anomalous exponent, $\alpha$, characterizes the nature of the anomalous diffusion as slower (sub-diffusion, $\alpha < 1$) or faster (super-diffusion, $\alpha > 1$) than Brownian motion (diffusive, $\alpha = 1$). Along microtubules, endosomal movement is powered by attachment to cytoplasmic dynein and various kinesin motor proteins^3,12,13^. Individual endosomes travel long intracellular distances ($\sim 1$–10 $\upmu$m) in short bursts of directed motility, interspersed with periods of diffusive^14,15^ and subdiffusive^9^ motion. It is likely that the heterogeneous character of endosome movement is influenced by cargo sorting and membrane fission^13–16^.

Ergodicity, i.e., the non-equivalence of time and ensemble averages, is one of the main tools that allow for selecting appropriate descriptions from various anomalous diffusion models^17,18^. These properties help to develop machine learning techniques for the classification of the diffusion model and the regression of the anomalous diffusion exponent of single-particle-trajectories^19,20^. The two main anomalous diffusion models are the ergodic fractional Brownian motion (FBM) and the non-ergodic continuous time random walk (CTRW). Many anomalous transport processes in living cells demonstrate ergodicity breaking^21–23^. Ergodicity breaking could stem from the non-stationary nature of the process, e.g., in the CTRW model when the distribution of the times associated with immobilization events has a divergent mean^17,24^. Several experiments suggested that nonergodic behaviour is a consequence of CTRW-type motion that may emerge as a result of interactions with cellular components^22,23,25^. Ergodicity breaking could also emerge as a consequence of the heterogeneity of the system, e.g., a heterogeneous ensemble of particles^26^, or heterogeneity within single trajectories (switching between motion with different diffusion coefficients)^27–29^. Conversely, the nonergodicity could mimic inhomogeneity in single particle trajectories^30^. In some systems, ergodic and non-ergodic processes coexist, e.g., in plasma membranes^31^, intracellular transport of insulin granules^32^, the motion of myosin motors in filament networks^33^, and the motion of sodium channels on the surface of hippocampal neurons^34^.

Recently we showed how space-time heterogeneity influences the anomalous transport of endosomes inside eukaryotic cells^35^. Instead of a single constant anomalous exponent and diffusion coefficient, the anomalous diffusion of endosomes was described by distributions of anomalous exponents and diffusion coefficients. We found an exponential probability distribution of anomalous exponents, $\alpha$, a power-law probability distribution of local generalized diffusion coefficients, *D*, and a power-law probability distribution of displacements, *X*, and displacement increments, $\Delta X$, for the endosomes. We showed that endosomal movement is ergodic and well described by superstatistical ensemble^36,37^ of space-time heterogeneous FBM (hFBM).

Although the time-space heterogeneity (the heterogeneity which is contained within single trajectories as they move through time and space) was very successful in describing many experimental features of endosomal dynamics such as distributions of displacements and increments, velocity autocorrelation functions and super-diffusive regime of the mean squared displacements at intermediate time scale, the time-space heterogeneous model was not able to describe the long-time sub-diffusion^35^. Instead, the time-space heterogeneous model predicted $t^2$ growth of the MSD at longer time scales. Here we show that the surprising aging behaviour is also not captured by the time-space heterogeneous model (see below). Therefore, something is missing in the full picture and the time-space heterogeneity alone is not enough to describe endosomal dynamics.

The main result of our present work is that, in addition to the time-space heterogeneity, there exists another type of heterogeneity that stems from differences among individual endosomes. We call it ensemble heterogeneity. To explain all experimental observables of endosomal dynamics one must consider the ensemble heterogeneity and the time-space heterogeneity together. Analysis of statistical ageing, defined as the dependence of quantities on both the measurement time *t* and the ageing time $t_a$^38^, provides another test of ergodicity^17^, since systems that show ageing also demonstrate the breaking of ergodicity. However, here we find that endosomal dynamics is ergodic but shows ageing. This paradox is explained by the heterogeneity of endosomal ensembles which we found in addition to the space-time heterogeneity within a single trajectory^35^. Thus we show that the ensemble heterogeneity mimics ageing.

## Results

We used two datasets of endosomal motion in 2D produced in different experiments using different cell lines and different cameras: (1) 103,361 trajectories of early endosomes in a stable MRC5 cell line (fibroblasts isolated from the lung tissue) expressing GFP-Rab5, obtained from tracking wide-field fluorescence microscopy videos (see Ref.^9^ for experimental details); (2) 7024 trajectories of GFP-Rab5-labelled endosomes in transiently transfected retinal pigment epithelium (RPE) cells (see Ref.^14^ for details).

**Figure 1:**
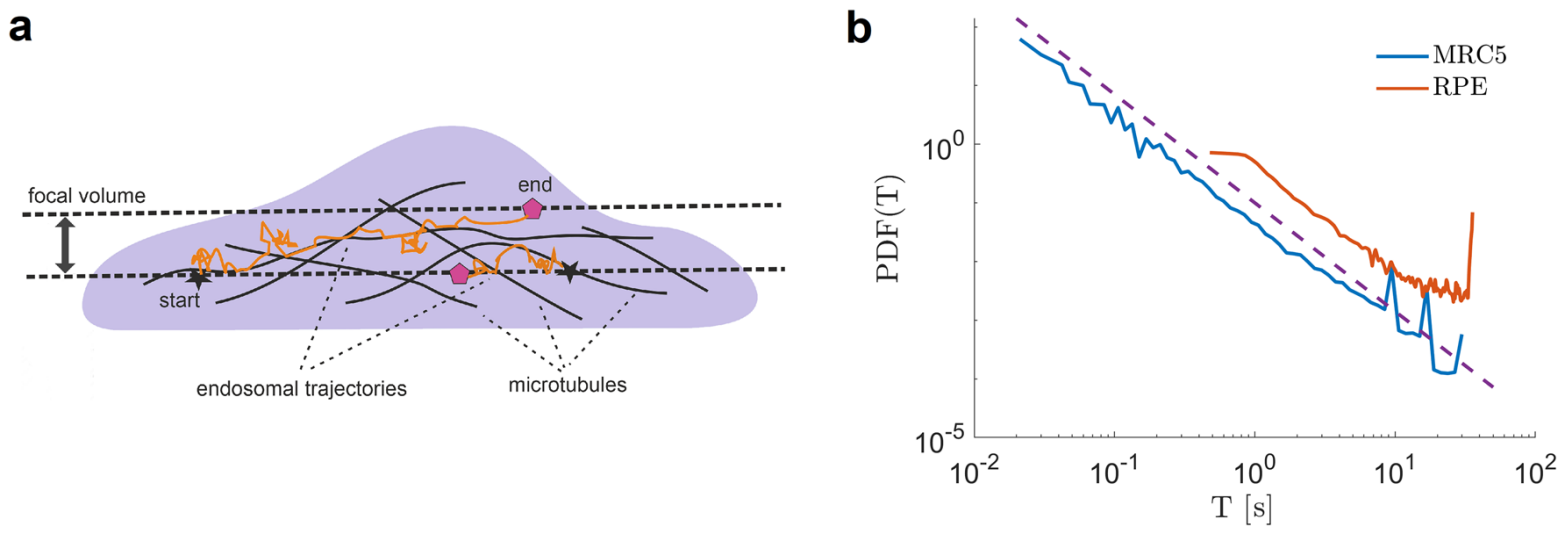
Heterogeneous dynamics of endosomes is characterized by random durations of their trajectories. (**a**) Schematic illustration of the cell (side view) where endosomal trajectories are recorded in the focal volume (between two horizontal dashed lines). Endosomes randomly enter and exit the focal volume where they move in a mesh of microtubules (black curves) randomly attaching to them. (**b**) The PDF of the duration *T* for trajectories in MRC5 and RPE cells. The dashed line is a power-law function $\sim T^{-2}$ to guide the eyes.

Thus, we compare the results of the analysis for two otherwise very different cell lines. Endosomes randomly enter and exit the focal volume (Fig. 1a) therefore the duration of their trajectories, *T*, was random. The PDFs of the duration of trajectories followed a power-law $PDF(T) \sim T^{-2}$ for both MRC5 and RPE cells (Fig. 1b).

### Endosomal dynamics is anomalous and ergodic

The mean squared displacements calculated either over an ensemble (ensemble-averaged MSD, eMSD) or using individual trajectories (time-averaged MSD, tMSD$_i$, here the subscript *i* stands for a trajectory’s index) are commonly used observables to characterize the dynamics. Ergodicity is defined as the equivalence of ensemble averages and long-time averages of individual endosome trajectories^17,39^:

1 $$\begin{aligned} \lim _{t \rightarrow \infty } \text{ tMSD}_i(t) = \text{ eMSD }(t). \end{aligned}$$

In experiments, however, often only short trajectories are available and it is impossible to check whether Eq. (1) holds. Therefore, the long time limit of $\text{ tMSD}_i(t)$ is replaced by additional averaging over the ensemble of trajectories to improve statistics,

2 $$\begin{aligned} \text{ e-tMSD }(t) = \left\langle \text{ tMSD}_i(t) \right\rangle = \text{ eMSD }(t). \end{aligned}$$

**Figure 2:**
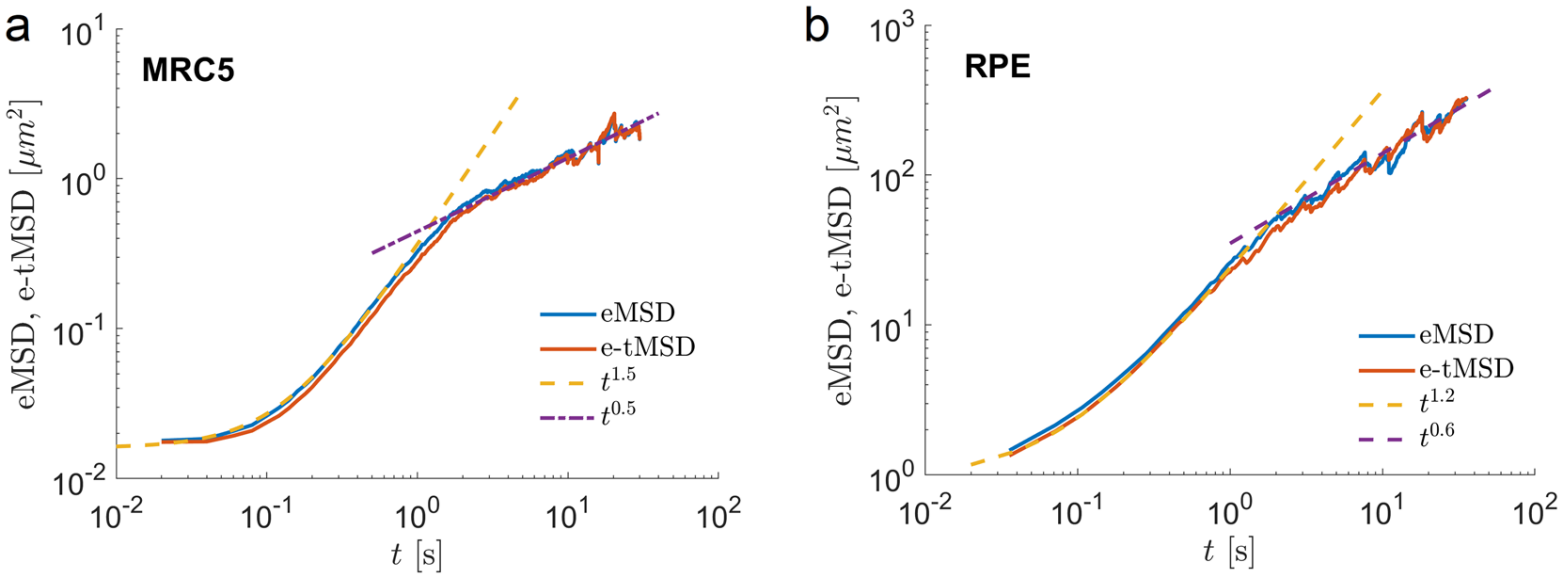
Diffusion of endosomes is anomalous and ergodic. (**a,b**) The ensemble-averaged MSD (eMSD) as a function of time *t* coincides with the ensemble-time-averaged MSD (e-tMSD) and shows the transition from superdiffusion (the dashed lines) to subdiffusion (the dotted lines).

Comparing eMSD with tMSD of experimental trajectories allow for discrimination between ergodic and non-ergodic behaviour and helps to select the appropriate diffusion model^17^. For MRC5 and RPE cells, eMSD and e-tMSD (tMSD after additional ensemble averaging) of experimental trajectories coincide indicating ergodic behaviour (Fig. 2a, b).

The eMSD is fitted to a power law function and the ensemble anomalous exponent $\alpha$ and the ensemble generalized diffusion coefficient $D_{\alpha }$ are extracted:

3 $$\begin{aligned} \text{ eMSD }(t) = 4 D_{\alpha } t^{\alpha }. \end{aligned}$$

Notice that $\alpha$ and $D_{\alpha }$ characterize the averaged transport properties of an ensemble of endosomal trajectories. At a small time scale $t<0.1$ s, the endosomal motion is subdiffusive. At time scales $0.2 \,\, \text {s}< t < 2$ s, molecular motors make the growth of EMSDs superdiffusive with the anomalous exponent $\alpha \sim 1.5 \pm 0.1$ and the ensemble generalized diffusion coefficient $D_{\alpha } \sim 0.08 \pm 0.01$ $\mu$m$^2/s^{\alpha }$ for endosomes in MRC5 cells and $\alpha \sim 1.2 \pm 0.1$ with $D_{\alpha } \sim 5.2 \pm 0.1$ $\mu$m$^2/s^{\alpha }$ for endosomes in RPE cells. The static localization error was estimated to be $\sigma = 90$ nm for endosomal trajectories in MRC cells and 300 nm in RPE cells. The superdiffusive behaviour of e-tMSD allows us to exclude the CTRW model which has characteristic linear growth of the tMSD^17^. At longer time scales ($t > 2$ s), the MSDs become apparently subdiffusive with $\alpha \sim 0.5$–$0.6 \pm 0.1$. Regardless of the diffusive regime, the ensemble-averaged eMSD in both MRC and RPE cells stays close to e-tMSD which indicates ergodicity of the endosomal motion.

**Figure 3:**
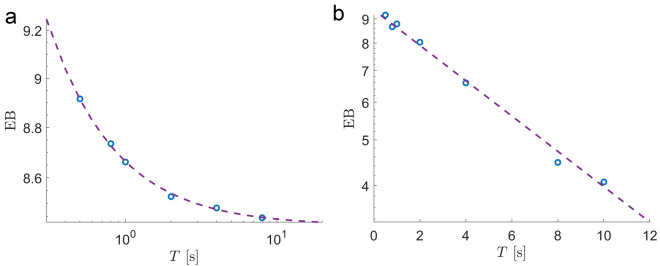
Ergodicity of ensembles of trajectories. Ergodicity breaking parameter EB, $\text{ EB }(t,T)= \left\langle \zeta ^2 \right\rangle - 1$, defined as the tMSD amplitude scatter $\zeta (t)=\text{ tMSD}_i(t)/\text{e-tMSD }(t)$ for trajectories of the duration *T*, as a function of *T*, calculated for all trajectories in MRC5 cells (**a**) and discarding trajectories shorter than *T* seconds (**b**). The dashed lines correspond to power law $8.4 + 0.25/T$ and exponential $0.94 \exp (-0.086 T)$ functions for (**a**) and (**b**) respectively.

**Figure 4:**
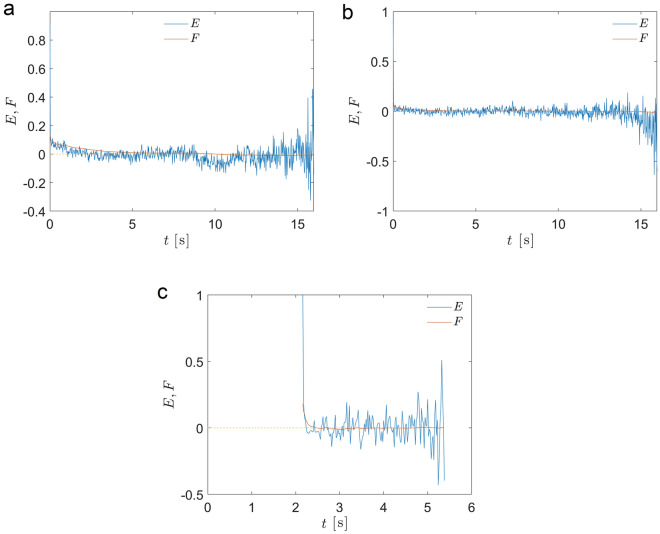
Testing ergodicity for single trajectories. Ergodicity, E, and the mixing, F, estimators defined in Ref.^44^ as functions of time for three sample trajectories (**a–c**).

The ergodicity breaking parameter, $\text{ EB }(t,T)= \left\langle \zeta ^2 \right\rangle - 1$, defined as the amplitude of the scatter of the tMSD $\zeta (t)=\text{ tMSD}_i(t)/\text{e-tMSD }(t)$ for trajectories of duration *T*, serves as an indicator of ergodicity^17,40,41^. For Brownian motion, EB tends to zero as EB$\sim t/T$ and shows a more complex power-law decay for FBM, EB$\sim (t/T)^{\beta }$, with the exponent $\beta (H)$ that depends on the Hurst exponent *H*^41^. In contrast, for CTRW and nonergodic heterogeneous diffusion processes, EB attains finite values at $t/T \rightarrow 0$^17,40,42^. We find that for endosomal dynamics EB also converges to a constant value. However, for the subset of trajectories with a duration of at least *T* seconds (e.g., discarding shorter trajectories), EB decays to zero as it should be for the ergodic process albeit in an exponential manner, EB$\sim \exp (-T)$ (Fig. 3). Ergodicity could be also checked on a single trajectory level^43,44^. Following^44^, we find that ergodicity, E, and the mixing, F, estimators converge to zero indicating the ergodicity of single trajectories (Fig. 4).

**Figure 5:**
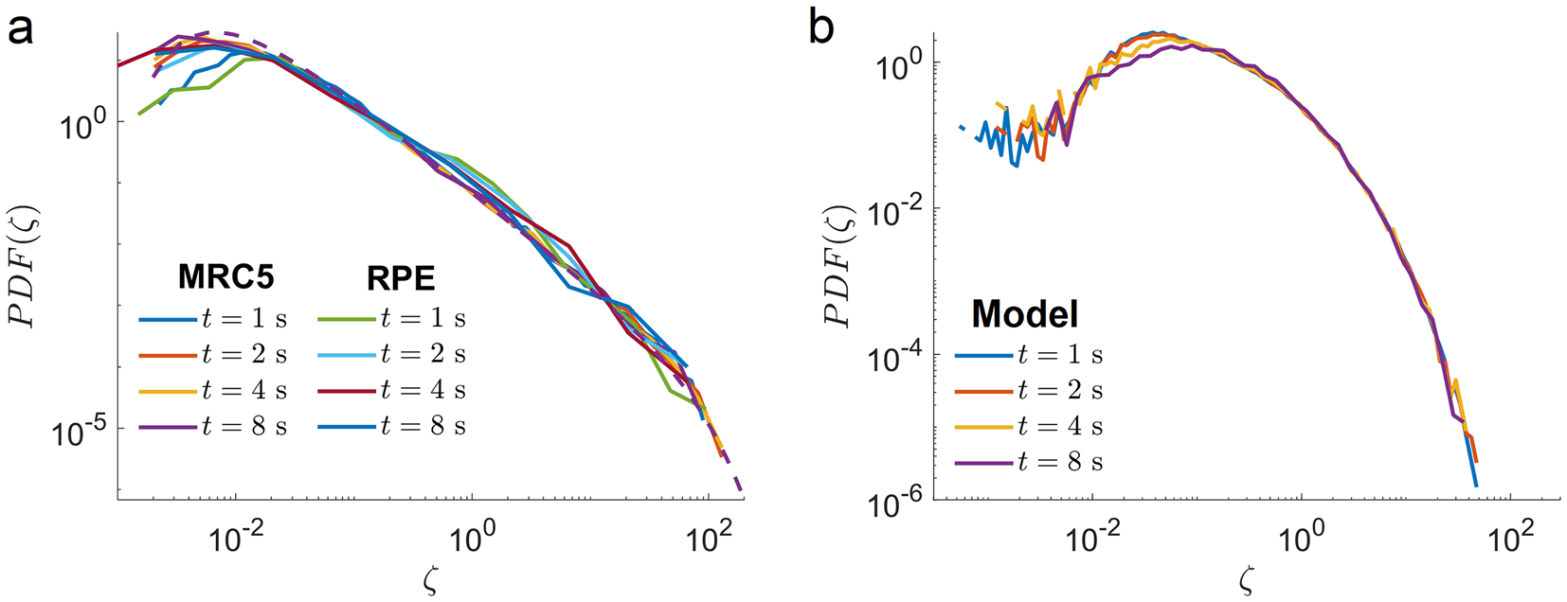
Distributions of the tMSD amplitude scatter $\zeta (t)$ for trajectories in MRC5 and RPE cells and for simulated trajectories of ensemble heterogeneous FBM model are broad. $PDF(\zeta )$ of $\zeta (t)=\text{ tMSD}_i(t)/\text{e-tMSD }(t)$ calculated at different time *t* (shown in the figure) for MCR5 and RPE trajectories (**a**) and trajectories of the ensemble heterogeneous FBM model (**b**). The dashed curve in (**a**) corresponds to a generalised Gamma distribution PDF$(\zeta ) \sim \zeta ^{\nu -1} \exp {(-a/\zeta -\zeta /b)}$ with $a=0.018$, $b=0.01$ and $\nu =1.5$.

Next, we calculated the distribution of the tMSD amplitude scatter $\zeta (t)$ for fixed *t* (Fig. 5). PDF($\zeta$) is often used to distinguish between ergodic and non-ergodic behaviour even for short-duration trajectories^45^. These PDFs are well described by the broad generalised Gamma distribution PDF$(\zeta ) \sim \zeta ^{\nu -1} \exp {(-a/\zeta -\zeta /b)}$ with three parameters *a*, *b* and $\nu$ (Fig. 5). The generalised Gamma distribution of $\zeta$ was found for ergodic processes such as FBM^46^ and also for the non-ergodic heterogeneous diffusion process with a space-dependent diffusion coefficient^42^. Notice that we found the broad distribution of $\zeta$ for ergodic heterogeneous ensemble of endosomes.

### Endosomes show ageing behaviour

Since we have found that for endosomal movement the ensemble and time-averaged MSD are equivalent (Fig. 2a,b), we expect no aging in endosomal dynamics. Surprisingly, we find that the MSDs of endosomes do show aging behaviour.

**Figure 6:**
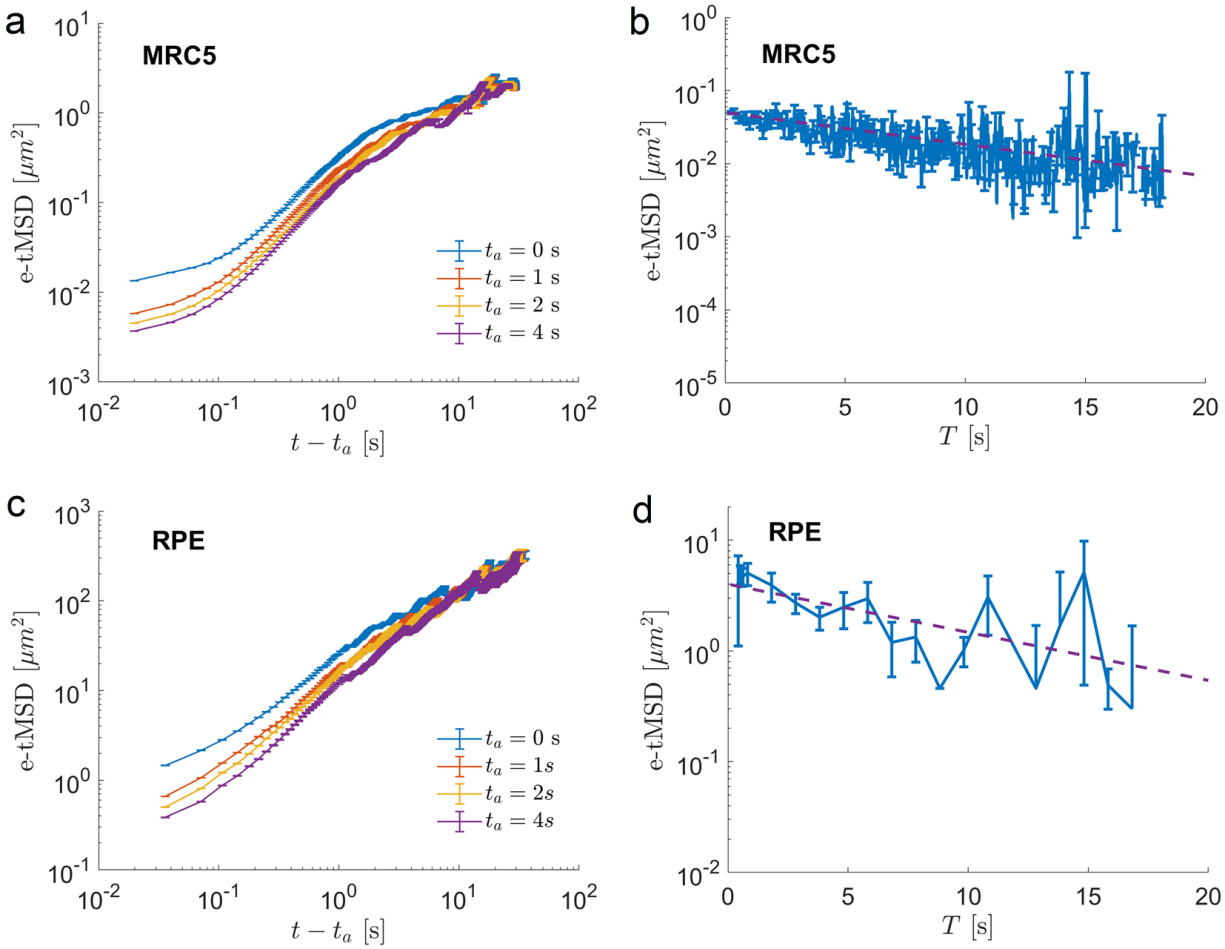
Diffusion of endosomes shows ageing behaviour. (**a,c**) e-tMSD as a function of time *t* show clear dependence on the aging time $t_a$. Different curves correspond to e-tMSDs calculated for different aging times. The error bars correspond to standard errors. (**b,d**) The e-tMSDs as functions of the duration of trajectories, *T*, calculated at $t=0.2$ s. The error bars represent the standard error of the mean. The dashed lines are fit by the exponential function $a \exp (-b T)$ with parameters a, b and their 95% confidence intervals given by $[0.0458\pm 0.0021, 0.0987\pm 0.0074]$ for (**b**) and $[4.9189\pm$1.5337, 0.1423$\pm 0.0872]$ for (**d**) .

The statistical aging is probed by delaying the start of the calculation of an observable by the aging time $t_a$ from the initiation of the process $t=0$. That is we are discarding $t_a$ seconds worth of data from each trajectory. In Fig. 6a, c the eMSDs of endosomes show a clear dependence on the aging time, $t_a$. We ensure that this behaviour is not due to the decreasing number of longer trajectories by performing simulations of FBM trajectories of the same duration as in the experiment. As expected, we observed no aging (data not shown). Ageing is also reflected in the decrease of e-tMSDs with the measurement time *T* (Fig. 6b, d). However, in contrast to ageing in the CTRW model, we find an exponential decay rather than a power law.

Clearly, ageing is incompatible with ergodicity. The question is how to reconcile these seemingly paradoxical behaviours? Below we show that the ensemble heterogeneity mimics the observed aging behaviour removing the contradiction. But first we test the transition from superdiffusion to subdiffusion (Fig. 2a,b) which reveals the dependence of diffusion coefficients on the duration of trajectories or the ensemble heterogeneity, and that the long-time subdiffusive regime is spurious.

### Endosomal long-time subdiffusion does not exist

Although transitions from superdiffusion to subdiffusion or progressively decreasing anomalous exponents are often observed, e.g., in the movement of micron-sized beads along microtubules^47^, endosomes filled with labeled nonviral DNA-containing polyplexes^5^, in single cell movement^48^, and in cytoplastic motion of passive nano-particles^49^, they are poorly understood.

**Figure 7:**
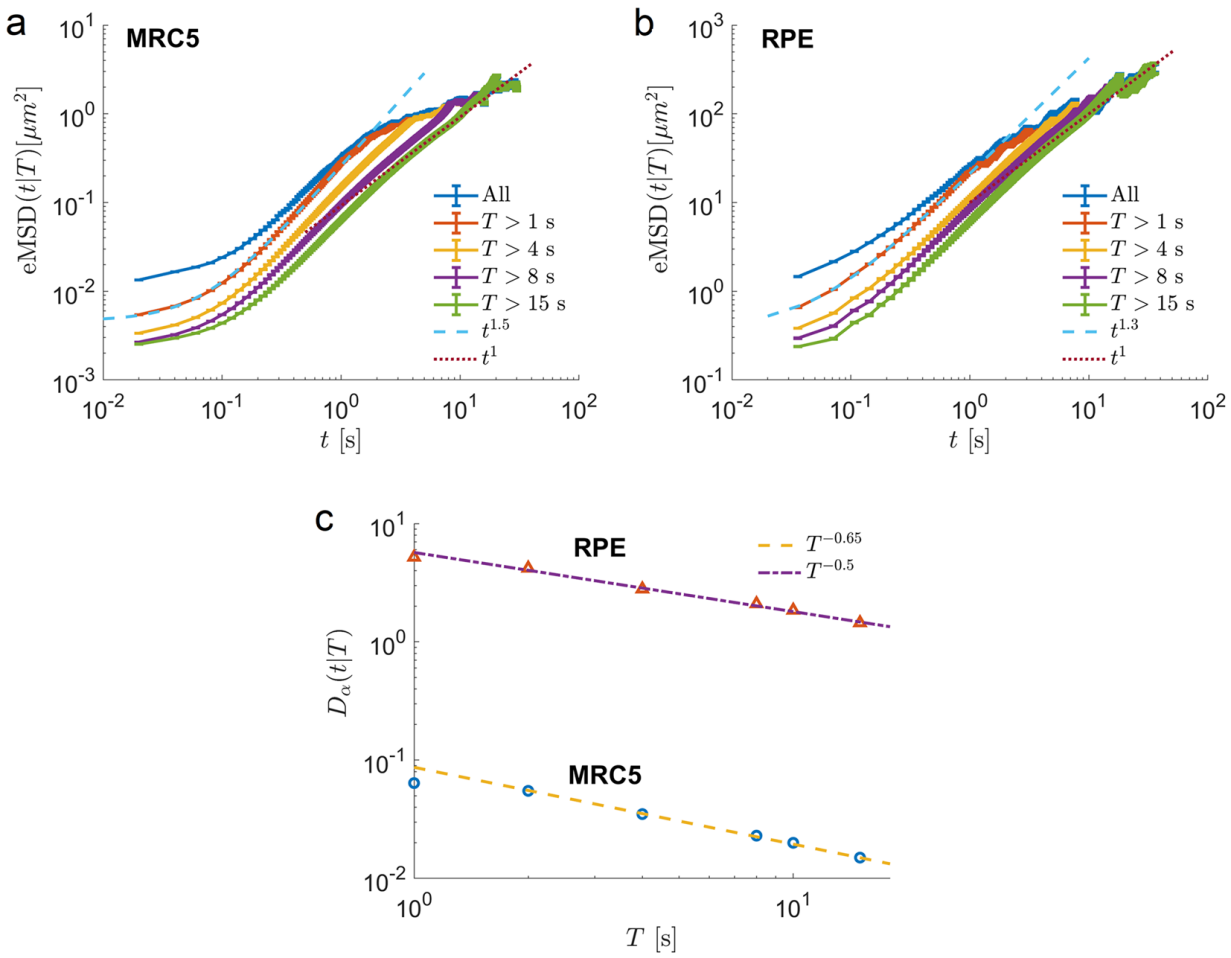
Ensemble of endosomes is heterogeneous as revealed by the dependence of the generalized diffusion coefficients on the trajectories duration. (**a,b**) Conditional eMSDs (eMSD calculated for the subsets of trajectories with a duration longer than *T* seconds) as functions of time *t* in MRC5 and RPE cells, respectively. Trajectories longer than 8 s (curves $T>8$ s and $T>15$ s) become diffusive at longer times. The dotted lines are linear functions to guide the eyes. (**c**) The numerical value of the conditional generalized diffusion coefficient, $D_{\alpha }(t|T)$, extracted from eMSDs in (**a**,**b**) by fitting to Eq. (6) , as a function of the minimal duration of trajectories *T*. The dashed and dashed-dotted lines correspond to power law functions $T^{-\gamma }$ with exponents $\gamma$ given in the figure. Notice that the units of the generalized diffusion coefficient are $[ \upmu \textrm{m}^2/\textrm{s}^{\alpha } ]$.

In order to test the transition from superdiffusion to subdiffusion in endosomal dynamics, we calculated the eMSDs for subsets of trajectories that have a duration of at least *T* seconds. That is, trajectories with a duration of less than *T* seconds were excluded. The results (Fig. 7a, b) show that the intermediate superdiffusive regime is conserved with the anomalous exponent $\alpha = 1.5 \pm 0.1$ for MRC trajectories and $\alpha = 1.2 \pm 0.1$ for RPE trajectories. However, the longer-time subdiffusive regime progressively disappears for $t<T$ s. From this observation, we conclude that the long-time subdiffusion regime is spurious and, in fact, does not exist. EMSDs of trajectories longer than 8 s (curves $T>8$ s and $T>15$ s) become diffusive at longer time scales with the anomalous exponent $\alpha \sim 1 \pm 0.1$. Thus, we show that the long-time subdiffusive regime in endosomal dynamics is spurious. Moreover, the superdiffusion-to-subdiffusion transition observed in other experiments should be carefully checked, as it may be artefactual. We stress that this is not due to imperfections in experimental procedures which favour short trajectories over long ones. Below we show that MSDs show the apparent subdiffusive scaling due to the heterogeneity of the ensemble of endosomes.

### Ensemble heterogeneity revealed by the dependence of $D_{\alpha }$ on the duration of trajectories

Notably, in Fig. 7a and b the subsets of longer trajectories have smaller conditional (on the duration of trajectories *T*) ensemble generalized diffusion coefficient $D_{\alpha }(t|T)$ since MSD curves are shifting downward for increasing *T*.

**Figure 8:**
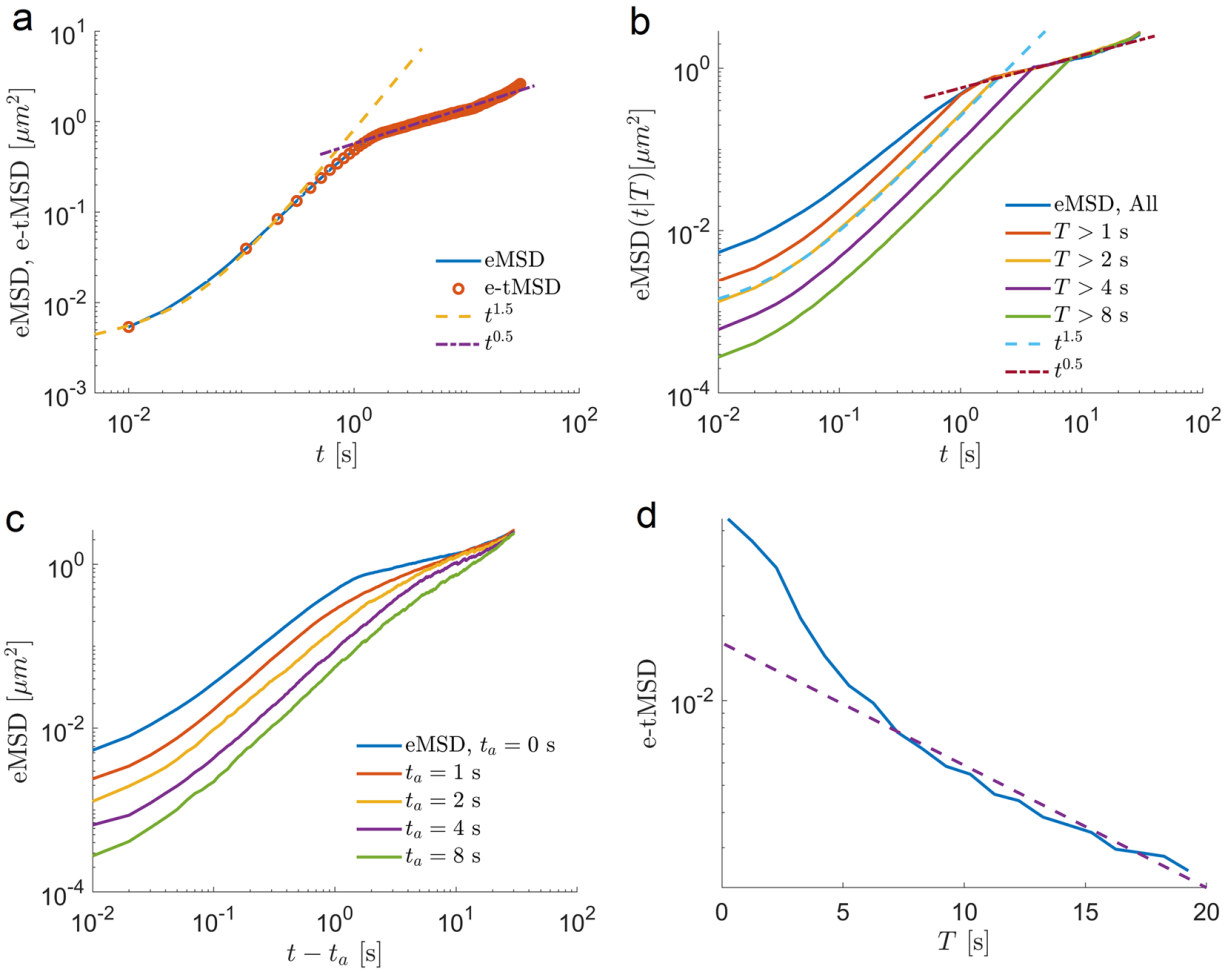
Simulations of a heterogeneous FBM model with diffusion coefficients that depend on the duration of trajectories capture the main features of endosomal dynamics. (**a**) eMSD and e-tMSD as functions of time *t*. EMSD coincides with e-tMSD indicating ergodicity. (**b**) Conditional eMSDs as functions of time *t* for the subsets of hFBM trajectories with a duration longer than *T* seconds. The dashed and the dashed-dotted lines are power-law fits. (**c**) hFBM model mimics ageing behaviour in ensemble MSDs  as a function of aging time (t-ta) and (**d**) in the decrease of e-tMSD as the measurement time, *T*, increases. The dashed line is the exponential function $\sim \exp (-0.1 T)$.

Fast endosomes with bigger diffusivities quickly leave the focus area and therefore have a shorter duration, while on the contrary, slowly moving endosomes with smaller diffusion coefficients stay longer in focus and have a longer duration^49^. This suggests that $D_{\alpha }$ depends on the duration of the observed trajectories or heterogeneity of endosomal ensembles. Early endosomes are known to fuse together and enlarge in size as they age in eukaryotic cells and move closer to the nucleus, which we expect will drive the power law slowing down of the generalized diffusion coefficient. To find the dependence of $D_{\alpha }(t|T)$ on the duration of trajectories, we extracted it by fitting the power laws to eMSD curves in Fig. 7a,b. $D_{\alpha }$ as a function of *T* (Fig. 7c) reveals the power law dependence on the duration of trajectories *T*, $D_{\alpha } \sim T^{-\gamma }$ with exponent $\gamma \sim 0.65$ for MRC trajectories and $\gamma \sim 0.5$ for RPE trajectories.

### Heterogeneity mimics ageing in eukaryotic cells

Once we established that heterogeneity leads to spurious subdiffusion at longer times, we wondered whether it can also explain the paradoxical ageing behaviour. To answer this question, we performed simulations of an ensemble heterogeneous FBM model (see Methods for details). Each trajectory was generated with a fixed anomalous exponent and fixed generalized diffusion coefficient. The key ingredient is the dependence of the diffusion coefficient on the duration of the trajectory as we found in the previous section. The ensemble heterogeneity in the FBM model was discussed in references^26,50^ but without the dependence of the diffusion coefficient on the duration of the trajectory. Notice that these models exhibit super weak ergodicity breaking in that Eq. (1) does not hold while Eq. (10) holds. This is in contrast to the weak ergodicity breaking of the CTRW model where both Eqs. (1) and (10) do not hold. While comparing the model with the experiments below, we ignore the super weak ergodicity breaking. After all, the infinite limit in Eq. (1) is not physical and most experimental trajectories are short.

The results of modelling (Fig. 8) reproduce all the main characteristics of heterogeneous endosomal dynamics. We recover the ergodic behaviour and the spurious subdiffusion regime which emerges at long time scales (Fig. 8a). This subdiffusive regime disappears for the subsets of longer trajectories (Fig. 8b). The intermediate superdiffusive regime is conserved and characterized by the same anomalous exponent but decreasing diffusion coefficient. This result implies that one cannot simply conclude the overall behaviour of the ensemble of endosomes just by examining the eMSD or the e-tMSD behaviour, since both are masked by the apparent subdiffusion at long time scales caused by the ensemble heterogeneity. Finally, the model reproduces the ageing behaviour of MSDs (Fig. 8c, d).

## Discussion

In this study, we investigated the endosomal motion of two distinct cell lines, MRC5 and RPE. Our analysis reveals that the endosomal dynamics of both cell lines exhibit anomalous and ergodic behavior. The ensemble MSD coincides with the time-averaged MSD and both show super-diffusive behaviour on the intermediate time scale and sub-diffusion at longer times. The ergodicity breaking parameter and the analysis of the ergodicity indicator of individual trajectories also confirm ergodicity. Since endosomes can be observed only in the focal region, their trajectories have random durations with a power-law distribution. Inspecting sub-ensembles of trajectories of different duration, we found that the long-time sub-diffusion is spurious. Surprisingly, we also discovered that endosomal dynamics exhibit statistical aging typical for systems that demonstrate the breaking of ergodicity. We provide a plausible explanation for this paradox by demonstrating the existence of heterogeneity within the endosomal ensembles, in addition to the space-time heterogeneity observed within a single trajectory. Our results suggest that the ensemble heterogeneity can mimic the aging behavior of the endosomal dynamics, thus contributing to the overall observed behavior. Overall, our findings highlight the importance of considering heterogeneity in understanding the dynamics of complex biological systems.

It is known for a long time that complex endosomal movement in eukaryotic cells is highly heterogeneous both in space and time. Trajectories consist of random switching from fast-directed movement powered by molecular motors interspersed with periods of slow movement characterized by anomalous diffusion, rather than Brownian motion^14^. Our recent results suggest that standard anomalous diffusion models which use a single constant anomalous exponent and a single generalized diffusion coefficient for the ensemble cannot adequately describe the transport of endosomes. The best model capable of doing so is time-space heterogeneous FBM^9,35^. The anomalous exponents were found to follow an exponential probability distribution while the generalized diffusion coefficients are described by a power-law probability distribution^9,35^. The time-space heterogeneous FBM model describes well experimental non-Gaussian distributions of displacements and displacement increments. However, the MSD behaviour, specifically the spurious long-time subdiffusive regime and ageing, were not captured by the time-space heterogeneity. Here we have shown that in addition to the time-space heterogeneity within a single trajectory, endosomes are characterized by ensemble heterogeneity. Ensemble heterogeneity based on FBM trajectories describes the spurious subdiffusion, displays ergodicity in the sense of equivalence (10) e.g., the equivalence of the ensemble and ensemble-time averaged MSDs and, at the same time, explains the paradoxical ageing behaviour. Recently it has been shown that the ensemble heterogeneity in random walks leads to strong memory effects and ballistic motion^51^.

The heterogeneity of endosomal transport has implications for sorting and delivering molecules for different biochemical reactions in different locations inside living cells. Rab5 early endosomes move towards the cell nucleus via dynein and fuse with one another and increase in size. This provides one biological mechanism for the power law slowing down of the generalized diffusion coefficient. It has a huge impact on the diffusion-limited reactions: it broadens the distribution of the first-passage time to a reaction event and increases the likelihood of both short and long trajectories hitting targets^14,44,52^. Both effects have a big impact on biochemical reaction rates, for example, signalling of Rab proteins on endosomes. The dynamic properties of other intracellular organelles, such as mitochondria and lipid droplets, share many similarities with endosomal transport^6–8,53^ and we anticipate their transport properties will also be heterogeneous.

## Methods

### Statistical analysis of experimental trajectories

The ensemble-averaged mean squared displacement (eMSD) of 2D experimental trajectories is defined, in its standard way, by:

4 $$\begin{aligned} \text{ eMSD }(t) = \left\langle (x_i(t)-x_i(0))^2 + (y_i(t)-y_i(0))^2 \right\rangle . \end{aligned}$$

The angled brackets denote averaging over an ensemble of trajectories, $\left\langle A \right\rangle =\sum _{i=1}^{N(t)} A_i/N(t)$, where *N*(*t*) is the number of trajectories present in the ensemble at time *t*. The eMSD is fitted by a power law function and the ensemble anomalous exponent $\alpha$ together with the ensemble generalized diffusion coefficient $D_{\alpha }$ are extracted:

5 $$\begin{aligned} \text{ eMSD }(t) = 4 D_{\alpha } t^{\alpha }. \end{aligned}$$

Since we are dealing with the ensemble of trajectories with random duration, we also introduce the conditional (on the duration of trajectories) ensemble MSD which is defined as eMSD calculated for a sub-ensemble of trajectories that have a duration longer than *T* seconds

6 $$\begin{aligned} \text{ eMSD }(t|T) = 4 D_{\alpha }(t|T) t^{\alpha }, \end{aligned}$$

where $D_{\alpha }(t|T)$ is the corresponding conditional generalized diffusion coefficient.

The time-averaged mean squared displacement (tMSD) of an individual trajectory $\{x_i, y_i \}$ of a duration *T* is defined by:

7 $$\begin{aligned} \text{ tMSD}_i(t) = \frac{ \int _{0}^{T-t} \left( x_i(t'+t)-x_i(t'))^2 + (y_i(t'+t)-y_i(t'))^2 \right) dt'}{T-t}. \end{aligned}$$

In experiments, trajectories could be short, thus the tMSDs of individual trajectories are averaged further over the ensemble of trajectories to get the ensemble-time-averaged MSD (e-tMSD):

8 $$\begin{aligned} \text{ e-tMSD }(t) = \left\langle \text{ tMSD}_i(t) \right\rangle . \end{aligned}$$

Similarly to the conditional ensemble MSD introduce in Eq. (6), we can introduce the conditional $\text{ tMSD}_{i}(t|T)$ and $\text{ e-tMSD }(t|T)$.

Ergodicity is defined as the equivalence of ensemble averages and long-time averages:

9 $$\begin{aligned} \lim _{t \rightarrow \infty } \text{ tMSD}_i(t) = \text{ eMSD }(t). \end{aligned}$$

In experiments, however, often only short trajectories are available and the long time limit of $\text{ tMSD}_i(t)$ is replaced by additional averaging over the ensemble of trajectories to improve statistics,

10 $$\begin{aligned} \left\langle \text{ tMSD}_i(t) \right\rangle = \text{ eMSD }(t). \end{aligned}$$

Aging is probed by delaying the beginning of the measurement by the aging time $t_a$ from the initiation of the process at $t=0$ or equivalently aging the system in $(-t_a,0)$ and starting the measurement at $t=0$. Statistical aging is defined as the dependence of quantities on both the measurement time *t* and the aging time $t_a$. The ensemble MSD for aging is:

11 $$\begin{aligned} \text{ eMSD }(t,t_a) = \int _{-\infty }^{\infty } r^2 P(r,t,t_a) \, dr = \left\langle (r_i(t+t_a)-r_i(t_a))^2 \right\rangle . \end{aligned}$$

Similarly, the aging tMSD of a single trajectory is defined as

12 $$\begin{aligned} \text{ tMSD}_i(t,t_a) = \frac{1}{T' -t} \int _{t_a}^{t_a+T'-t} \left( r_i(t'+t)-r_i(t'))^2 \right) dt'. \end{aligned}$$

Notice that for experimental trajectories, the duration of a trajectory, $T'$, must be adjusted as $T'=T-t_a$, where *T* is the duration of the non-aged trajectory. One can omit such an adjustment when dealing with simulated trajectories which can be made as long as required, but not in typical experiments.

### Simulation of ensemble heterogeneous FBM model

We simulated trajectories of the heterogeneous FBM with the discrete Langevin equation $\{X,Y\}(t) = \text{ FBM}_1(t)$ where $\text{ FBM}_1(t) = \{\text{ FBM}_{1,x}, \text{ FBM}_{1,y}\}$ are independent fractional Brownian motions generated using the ffgn function in Matlab (Natick, MA) with constant anomalous exponent $\alpha _1=0.75$ and constant generalized diffusion $D_1$ coefficient for each trajectory. $D_1$ depends on the duration of a trajectory which was chosen as follows. Since endosomes randomly enter and exit the focus volume, the duration of trajectories is random and drawn from the distribution $\text{ PDF }(T) \sim T^{-2}$. The diffusion coefficients were chosen as a decreasing function of the duration of trajectories. Specifically, $D_1 = 0.25 T^{-0.1}$ for $T<2$ s and $D_1 = 0.5 T^{-1.15}$ for $T>2$ s. Notice that the power law of the dependence of $D_1$ on *T* is not the same as we observed in the experiments (see the main text). This is because, for experimental trajectories, we determined the dependence of the generalized diffusion coefficient on the duration of trajectories for the sub-ensembles of trajectories with a duration longer than *T* s. Due to the lack of statistics, it was not possible to determine the dependence of $D_1$ for trajectories of an exact duration *T* s which we used to set up the model. Also, for the sake of the simplicity of the model, $D_1$ was chosen to be constant for a given trajectory, while for experimental trajectories it also changes along the trajectory as we have found in our previous work (see Ref. [31] in the main text). Despite this simplification, the model is capable of explaining all the main experimental features discussed in this paper.

In order to reproduce the subdiffusive behaviour at a shorter time scale ($t<0.1$ s), we used another FBM motion (which has the same duration as FBM$_1$) with a constant anomalous exponent $\alpha _2=0.1$ and constant generalized diffusion coefficient $D_2 = 0.009$. Thus, we model the endosomal dynamics as $\{X,Y\}(t) = \text{ FBM}_1(t) + \text{ FBM}_2(t)$. Notice that the ergodic behaviour of experimental trajectories, e.g. eMSD$=$e-tMSD, rules out the measurement noise as the origin of the observed subdiffusion for $t<0.1$ s. Indeed, if we consider the measurement noise as additive and Gaussian, $\{X,Y\}(t) = \text{ FBM }(t) + \{\xi _x, \xi _y\}$ where $\{\xi _x, \xi _y\}$ are Gaussian iid random variables with zero mean and variance $\sigma _x=\sigma _y=\sigma$, the ensemble MSD of $\{X,Y\}(t)$ is easily calculated as eMSD(t)$= 4 D t^{\alpha } + 2 \sigma ^2$ and the time-averaged MSD reads e-tMSD(t)$= 4 D t^{\alpha } + 4 \sigma ^2$. While one recovers the ergodic long-time behaviour, at a shorter time scale, the additive noise makes eMSD and tMSD different. This contradicts our experimental findings for endosomal dynamics shown in the main text Fig. 1a, d and rules out the measurement noise as an origin of the short-time subdiffusion.

## Data Availability

The data that support the findings of this study are available from the corresponding author upon reasonable request.
